# Inexpensive *Ipomoea aquatica* Biomass-Modified Carbon Black as an Active Pt-Free Electrocatalyst for Oxygen Reduction Reaction in an Alkaline Medium

**DOI:** 10.3390/ma8105331

**Published:** 2015-09-25

**Authors:** Yaqiong Zhang, Chaozhong Guo, Zili Ma, Huijuan Wu, Changguo Chen

**Affiliations:** 1 College of Chemistry and Chemical Engineering, Chongqing University, Chongqing 400044, China; 20121802076@cqu.edu.cn (Y.Z.); czguo@cqwu.edu.cn (C.G.); mzl@cqu.edu.cn (Z.M.); 20131802094@cqu.edu.cn (H.W.); 2 Research Institute for New Materials Technology, Chongqing University of Arts and Sciences, Chongqing 402160, China

**Keywords:** oxygen reduction reaction, Pt-free, *Ipomoea aquatic*, biomass

## Abstract

The development of inexpensive and active Pt-free catalysts as an alternative to Pt-based catalysts for oxygen reduction reaction (ORR) is an essential prerequisite for fuel cell commercialization. In this paper, we report a strategy for the design of a new Fe–N/C electrocatalyst derived from the co-pyrolysis of *Ipomoea aquatica* biomass, carbon black (Vulcan XC-72R) and FeCl_3_·6H_2_O at 900 °C under nitrogen atmosphere. Electrochemical results show that the Fe–N/C catalyst exhibits higher electrocatalytic activity for ORR, longer durability and higher tolerance to methanol compared to a commercial Pt/C catalyst (40 wt %) in an alkaline medium. In particular, Fe–N/C presents an onset potential of 0.05 V (*vs*. Hg/HgO) for ORR in an alkaline medium, with an electron transfer number (*n*) of ~3.90, which is close to that of Pt/C. Our results confirm that the catalyst derived from *I. aquatica* and carbon black is a promising non-noble metal catalyst as an alternative to commercial Pt/C catalysts.

## 1. Introduction

Several studies have aimed to discover a large reserve of renewable and environment-friendly alternative energy sources, because of worsened environmental pollution and energy shortage. Fuel cells (FCs) are novel efficient, reliable, environment-friendly and flexible electric power generators, which can directly convert chemical energy into electrical energy with high conversion efficiency. The products of FC power generators, namely carbon dioxide and water, are nonhazardous to the environment. However, these power generators present slow kinetics of the cathodic oxygen reduction reaction (ORR) and therefore require the use of catalysts. Currently, the most effective and widely-used ORR electrocatalysts are those based on Pt. Nevertheless, Pt-based electrocatalysts are expensive and present poor methanol tolerance and low abundance, thereby hindering their commercialization [1,2,3,4,5]. In this regard, non-noble electrocatalysts with good catalytic activity, excellent methanol tolerance and high yield for FCs must be explored to replace Pt-based catalysts [6,7,8,9].

In 1964, Jasinski [10] reported that the transition metal macrocyclic cobalt phthalocyanine exhibits electrocatalytic activity for ORR. Since then, N-containing transition metal macrocyclic compounds have attracted considerable attention as promising alternatives to Pt-based ORR catalysts in FCs. Several scientists have investigated the oxygen-reduction catalytic activity of inexpensive electrocatalysts by using different N-containing carbon materials as effective precursors [11,12,13]. These carbon materials include crude biomass, such as hemin [14,15,16,17,18], *Typha orientalis* [19], monkey grass [20], soybeans [21] and soya milk [22], which have attracted considerable interest, because they are abundant, environment friendly and inexpensive [23,24,25,26,27]. All of these studies have focused on using natural porphyrinato complexes containing hemin, *T. orientalis* and monkey grass to form catalytic sites.

*Ipomoea aquatica*, commonly known as swamp morning glory, is a widely-cultivated vegetable in the rural area of southern China. *I. aquatica* leaves contain abundant chlorophyll and Mg porphyrins, and the latter can be used as a precursor for the development of inexpensive ORR catalysts.

In this work, Fe–N/C catalyst was synthesized through a simple and readily-scalable approach by using *I. aquatica* leaves as the precursor. This approach comprises facile acid leaching and post-treatment in nitrogen gas (N_2_) with Fe salts and carriers. The fabricated Fe–N/C catalyst exhibits excellent catalytic activity and 4e-pathway selectivity toward ORR, durability and methanol tolerance in an alkaline medium.

## 2. Experimental Section

### 2.1. Chemicals and Reagents

All of the reagents were of analytical grade and used without further purification. *I. aquatica* was obtained from a vegetable market in Chongqing, China, and was washed with distilled water before crushing. N_2_ (99.9%) was purchased from Shang Yuan Company (Chongqing, China). Carbon black (Vulcan XC-72R) was acquired from Cabot^®^ (Cabot Corporation, Boston, MA, USA). Commercially available 40 wt % Pt/C catalyst (JM Pt/C 40 wt %) was purchased from Johnson Matthey Corp (Yantai, China).

### 2.2. Catalyst Preparation 

Typically, fresh leaves of *I. aquatica* (hereafter IA) were initially washed and dried at 90 °C for 36 h. The dried leaves were subsequently ground into powder and used as a starting material. The dried leaf powder was mixed with 80 mL of 0.5 M H_2_SO_4_ (Gai Tang Chemical Co., Ltd., Chengdu, China), and the obtained suspension was stirred and etched for 12 h. The product (denoted as Ipomoea aquatic after acid etching (IAA)) was then collected by centrifugation at 4500 rpm for 20 min to remove large-sized products and then dried at 90 °C for 9 h.

Briefly, 0.1 g of IAA was mixed with carbon black support (at a mass ratio of 1:1) and FeCl_3_·6H_2_O (Zhiyuan Chemical Products Co., Ltd., Zhengzhou, China) through ball milling for 30 min. The obtained substance (Fe–N/C) was treated in flowing N_2_ at 900 °C for 2 h.

For control, Fe–N/C was subjected to acid leaching using 5 M H_2_SO_4_ (Gai Tang Chemical Co., Ltd., Chengdu, China) solution at room temperature for 24 h. The as-prepared sample was marked as N/C (Fe). N/C was obtained through heat treatment of IAA and the carbon black support at the same mass ratio without FeCl_3_·6H_2_O.

### 2.3. Characterization

Electrochemical measurements were conducted on a CHI 660 electrochemical workstation (CH Instruments, Austin, TX, USA), with a common three-electrode electrochemical cell. A platinum wire and an Hg/HgO (1 M KOH (Heng Aode Technology Co., Ltd., Beijing, China)) electrode were used as counter and reference electrodes, respectively. A rotating disk electrode (RDE) with a glassy carbon (GC) electrode (5-mm diameter, Tianjing Lanlike Electrochemical Instruments, Tianjin, China) coated with catalyst ink was used as a working electrode. Typically, 10 μL of the catalyst suspension dispersed in 0.5 wt % Nafion/isopropanol solution (DuPont, Wilmington, DE, USA) were dropped onto the pre-polished GC disk surface and then dried at room temperature. Sample loading is *ca*. 10 mg·cm^−2^, and the mass loading of the commercial Pt/C-modified electrode is 5 mg·cm^−2^. All RDE experiments for ORR were performed within −0.7 to 0.3 V (*vs*. HgO/Hg) at a scan rate of 5 mV·s^−1^ in O_2_-saturated 0.1 M KOH solution.

X-ray photoelectron spectra (XPS) were obtained with a VG Escalab220 iXL spectrometer (VG Scientific, St Leonards-on-Sea, UK) fitted with an Al Kα (hν = 1486.69 eV) X-ray source to investigate surface composition and chemical status of the catalyst. X-ray diffraction (XRD) analysis was also conducted using a Shimadzu XRD-6000 X-ray diffractometer (Shimadzu, Kyoto, Japan) with Cu Kα_1_ radiation (λ = 1.54178 Å) at scan rate of 4° min^−1^.

## 3. Results and Discussion

**Figure 1:**
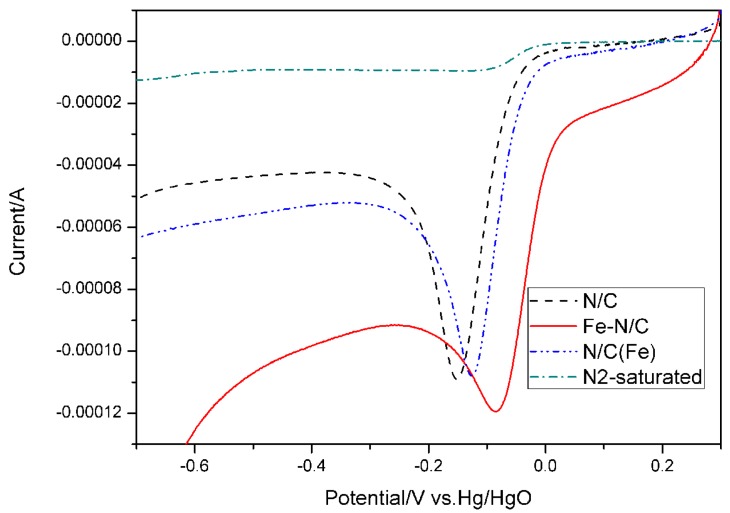
Linear sweep voltammograms of N/C, Fe–N/C and N/C (Fe) at a scan rate of 50 mV·s^−1^ in O_2_- and N_2_-saturated 0.1 M KOH solutions.

The electrocatalytic activities of N/C, Fe–N/C and N/C (Fe) for ORR in 0.1 M KOH solution saturated with O_2_, as well as Fe–N/C in N_2_-saturated 0.1 M KOH solution, are shown in Figure 1. The onset potential (*E*_onset_) and peak potential (*E*_p_) are used to evaluate the electrocatalytic activities of the samples. In the figure, the linear sweep voltammograms (LSVs) of N/C, Fe–N/C and N/C (Fe) in O_2_-saturated solution present well-defined cathodic peaks. Fe–N/C exhibits the highest positive *E*_onset_ and *E*_p_ values over N/C and N/C (Fe), and is thus regarded as the sample with the highest electrocatalytic activity. However, no characteristic peak curves are present in the LSV of Fe–N/C in N_2_-saturated 0.1 M KOH solution, which demonstrates the excellent electrocatalytic activity of Fe–N/C toward ORR.

The catalyst Fe–N/C exhibits higher catalytic activity than N/C because of the participation of Fe in the heat treatment. To investigate whether or not Fe is an integral part of the active site in the catalyst, we determined the catalytic activity of N/C and N/C (Fe). Metal elements do not participate in the preparation of N/C, whereas Fe is involved at the beginning of the pyrolysis of N/C (Fe), but soaked in acid after the process. The *E*_onset_ and *E*_p_ values of N/C (Fe) are similar to those of N/C, but shift negatively relative to those of Fe–N/C upon soaking out of Fe. These results indicate that Fe is an integral part of the efficient ORR catalyst, and Fe–N/C exhibits the highest electrocatalytic activity among the sample catalysts.

**Figure 2:**
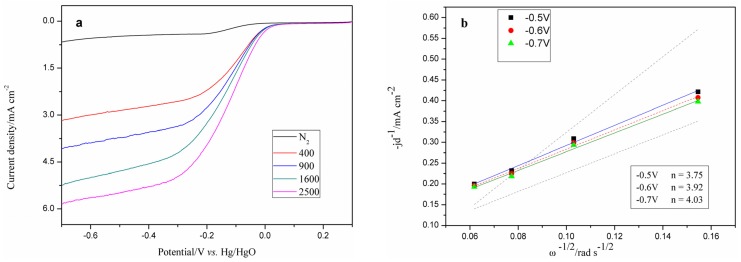
(**a**) Rotating disk electrode (RDE) voltammograms of the Fe–N/C electrode in O_2_-saturated 0.1 M KOH under different rotation rates at a scan rate of 5 mV·s^−1^. (**b**) Koutecky–Levich plots of the Fe–N/C electrode at different potentials.

RDE measurements were performed under different rotation rates to investigate the electrocatalytic mechanism of catalyst Fe–N/C for ORR and to determine the dominant processes in the alkaline medium (0.1 M KOH). Figure 2a shows that the current density on the Fe–N/C electrode increases with increasing rotation speed (from 400 to 2500 rpm). The overall number of transferred electrons per oxygen molecule (*n*) involved in the ORR process is determined by the Koutecky–Levich (K–L) Equation [26]:

(1) $$\frac{1}{j_{\text{d}}}=\frac{1}{j_{\text{k}}}+\frac{1}{0.62nFC_{\text{O}}D_{\text{O}}^{2/3}{\text{ν}}^{-1/6}{\text{ω}}^{1/2}}$$

where *j*_d_ is the diffusion-limited current density, *j*_k_ is the kinetic current density of the ORR, *F* is the Faraday constant (96,485 C·mol^−1^), *C*_O_ is the O_2_ saturation concentration in the aqueous solution (1.2 × 10^−6^ mol·cm^−3^), *D*_O_ is the O_2_ diffusion coefficient in 0.1 M KOH electrolyte (1.9 × 10^−5^ cm^2^·s^−1^), *ν* is the kinetic viscosity of the solution (0.01 cm^2^·s^−1^) and *ω* is the electrode rotation rate (rpm). Figure 2b shows the K–L plot of *j*_d_^−1^ *vs*. ω^−1/2^ for Fe–N/C.

The parallelism and linearity of the K–L plots indicate consistent electron transfer across different potentials and first-order reaction kinetics with respect to the dissolved oxygen concentration [28]. The *n* value for ORR of Fe–N/C is ~3.90 from −0.5 to −0.7 V *vs.* that of the HgO/Hg electrode. The obtained value is considerably close to four, which is the *n* value for the ORR of commercial Pt/C. ORR catalyzed by Fe–N/C involves a mixture of two- and four-electron transfer pathways, but is dominated by the latter to mainly produce H_2_O. O_2_ reduction via the 4e-pathway is highly desirable to obtain maximum energy capacity. These results prove that Fe–N/C is a suitable alternative cathodic catalyst to commercial Pt-based catalysts for FC applications with reduced cost.

The catalyst Fe–N/C exhibits excellent electrocatalytic activity with positive *E*_onset_ and *E*_p_ values for ORR. Nevertheless, stability and immunity toward methanol crossover are also required for practical applications. Pt-based catalysts demonstrate low stability and poor immunity toward methanol crossover. These limitations hindered the use of Pt-based catalysts in FCs [29].

**Figure 3:**
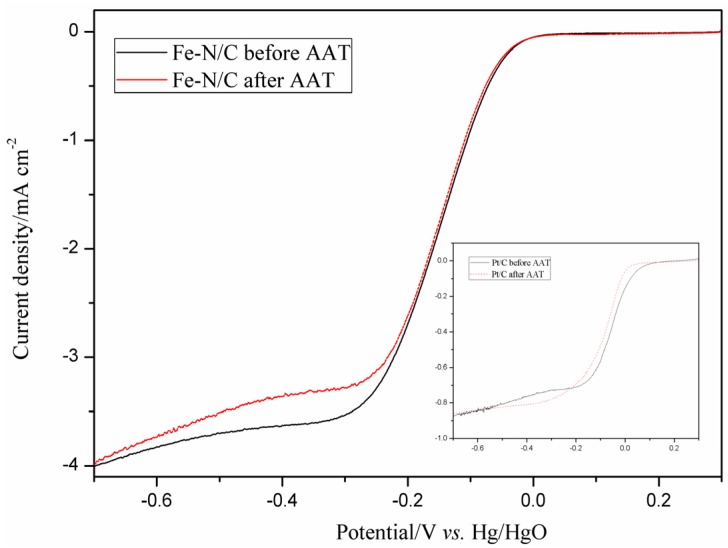
Oxygen reduction reaction (ORR) polarization curves for Fe–N/C and Pt/C (inset) before and after the accelerated aging test (AAT) in 0.1 M KOH solution at 1600 rpm.

The long-term stability of the Fe–N/C electrocatalyst was evaluated using an accelerated aging test (AAT) by continuous scanning 2000 cycles at a scan rate of 100 mV·s^−1^ and a potential range of −0.7 to 0.3 V (*vs*. Hg/HgO) in O_2_-saturated 0.1 M KOH solution. The ORR performance of Fe–N/C before and after AAT was assessed using the same three-electrode cell described in Section 2.3. For comparison, the stability of the JM Pt/C 40 wt % catalyst was also measured and is shown in the inset of Figure 3. The *E*_onset_ and half-wave peak (*E*_hw_) negatively shift by 9 and 13 mV at the Fe–N/C-coated electrode after AAT relative to those before AAT. Moreover, the *E*_onset_ and *E*_hw_ of the JM Pt/C 40 wt % catalyst negatively shift by 54 and 36 mV under the same condition. Thus, the catalyst Fe–N/C exhibits higher stability than JM Pt/C 40 wt %. 

**Figure 4:**
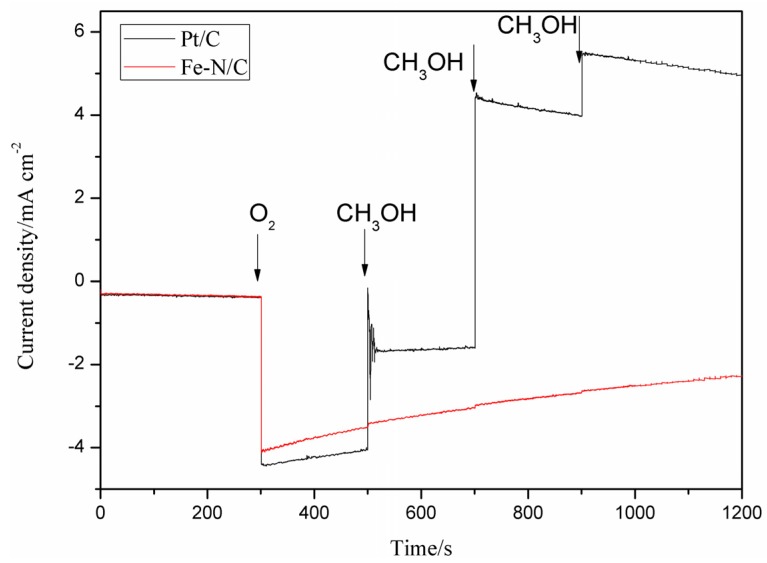
Amperometric current-time curves of Fe–N/C and Pt/C in N_2_-saturated 0.1 M KOH solution under magnetic stirring (600 rpm) and N_2_-protection from 0 to 300 s, followed by immediate introduction of O_2_. The applied potential is −0.1 V. The arrows indicate the sequential addition of 5.0 M methanol.

As shown in Figure 4, the catalyst JM Pt/C 40 wt % and Fe–N/C catalysts show an obvious cathodic current response upon the introduction of O_2_ into 0.1 M KOH solution. By contrast, a different phenomenon occurs when 5.0 M methanol (Chuandong Chemical Industry Co., Ltd., Chongqing, China) was added. The amperometric response of catalyst Fe–N/C remains stable with increasing amounts of methanol added to the solution at a time interval of 200 s. Meanwhile, catalyst JM Pt/C 40 wt % demonstrates a step-down decrease in cathodic current response with successive addition of methanol. These results confirm that the Fe–N/C catalyst exhibits significantly satisfactory immunity toward methanol.

Although catalyst Fe–N/C also exhibits slightly higher overpotential toward ORR than commercial catalyst JM Pt/C 40 wt %, the former contains an electron transfer number (*n*) similar to that of Pt/C and presents high tolerance to methanol and stability; hence, catalyst Fe–N/C can be used as an alternative active ORR catalyst with considerable potential for FC and metal-air battery applications.

**Figure 5:**
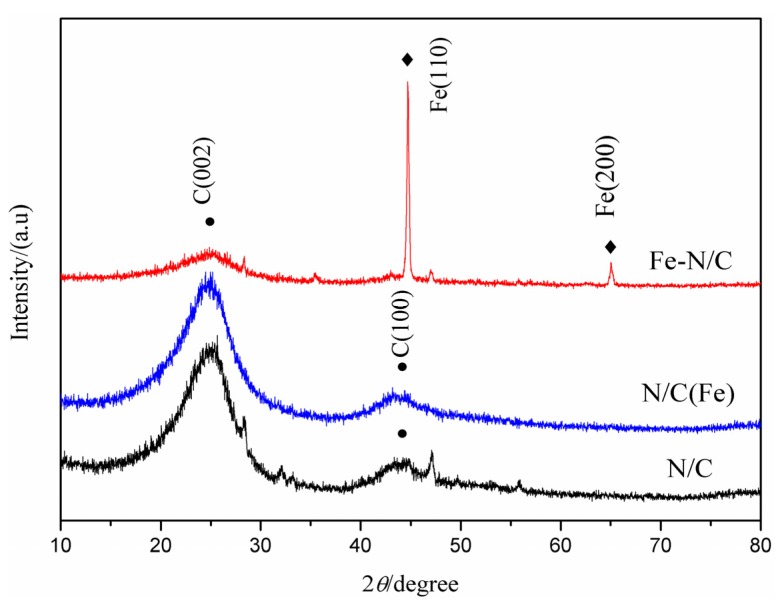
XRD patterns of N/C, Fe–N/C and N/C (Fe).

Figure 5 shows the XRD patterns of the three catalyst samples. The diffraction peaks at 2θ = 25° and 44° corresponds to the (002) and (101) planes of carbon, respectively. A crystal peak in catalyst Fe–N/C may be due to the incorporation of FeCl_3_·6H_2_O. The XRD test results of the three samples show that adding FeCl_3_·6H_2_O significantly affects the structure of the catalyst. Nevertheless, no diffraction peaks for Fe are found in catalyst N/C (Fe), which indicates that this element is mostly removed through acid leaching.

**Figure 6:**
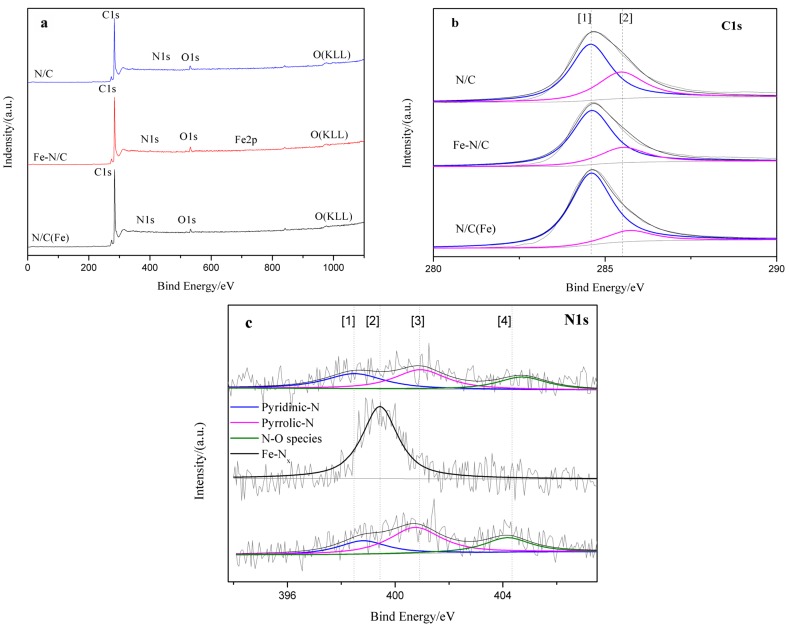
(**a**) XPS survey spectra of N/C, Fe–N/C and N/C (Fe). (**b**) XPS spectra of C1s of N/C, Fe–N/C and N/C (Fe). (**c**) XPS spectra of N1s of N/C, Fe–N/C and N/C (Fe).

The XPS measurements of the three catalysts were conducted to investigate surface composition and chemical status of the catalysts, and the results are shown in Figure 6. The XPS survey spectra of catalyst N/C, Fe–N/C and N/C (Fe) (Figure 6a) show C1s, O1s, N1, and less noticeable Fe2p signals (in Fe–N/C). Fe was not detected by XPS; nevertheless, this element could not be considered as absent from the material surface, because the XPS technique cannot accurately detect the presence of Fe because of its limited detection depth.

The C1s high-resolution spectra of the products are slightly asymmetric, as shown in Figure 6b. Each spectrum can be deconvoluted into two peaks, with a dominant peak at 284.5 eV and a small peak at 285.5 eV. The dominant peak is attributed to the *sp*^2^-hybridized C–C bond, and the small peak indicates the existence of functional groups, such as C–OH [30] and C–N configurations [20,31]. 

In the complex N1s high-resolution spectra (Figure 6c), pyridinic-N, Fe–N*_x_*, pyrrolic-N and oxidized pyridinic-N species are clearly doped. The peak at 398.6 ± 0.2 eV corresponds to pyridinic-N species, whereas that at 399.6 ± 0.2 eV is speculated as Fe–N*_x_* [32,33,34]; meanwhile, the peak at 400.8 ± 0.1 eV is ascribed to the pyrrolic-N structure [32,34], and that at 402–405 eV is due to oxidized pyridinic-N(N^+^–O^−^) species [32,33,35,36,37].

The appearance of the Fe–N*_x_* characteristic peak further verifies that the catalyst comprises Fe. With the high levels of Fe–N*_x_* and good ORR activity of Fe–N/C as bases, the following may be assumed: (1) N and Fe may have contributed to the excellent activity in alkaline solutions; and (2) Fe–N*_x_* may be an effective active site. Furthermore, the N/C (Fe) sample exhibits slightly higher ORR activity than N/C, and the content of pyrrolic-N in N/C (Fe) is higher than that in N/C (Table 1); therefore, we could deduce that pyrrolic-N may be responsible for ORR activity.

**Table 1:** Peak positions, areas, full widths at half maximum (FWHM) and concentrations of N1s species in N/C, Fe–N/C and N/C (Fe) obtained from the XPS results.

Catalyst	Peak	Area	FWHM	Content
N/C	398.497 eV	803	2.600 eV	35%
	400.936 eV	909	2.300 eV	40%
	404.734 eV	567	2.200 eV	25%
Fe–N/C	399.436 eV	2328	1.603 eV	100%
N/C(Fe)	398.803 eV	599	2.167 eV	25%
	400.743 eV	12159	2.304 eV	50%
	404.173 eV	612	2.050 eV	25%

## 4. Conclusions

Fe–N/C is synthesized through the co-pyrolysis of *I. aquatica* leaves, carbon black (Vulcan XC-72R) and FeCl_3_·6H_2_O under nitrogen atmosphere. Fe is an integral part of the active Fe–N/C catalyst site. Electrochemical results show that Fe–N/C is an active catalyst with a four-electron transfer pathway toward ORR and with good stability and immunity toward methanol crossover. All of the results have proven that the Fe–N/C catalyst can be a promising electrocatalyst with significant practical value.
